# Case Report: A rare presentation of rapidly progressive moyamoya disease refractory to unilateral surgical revascularization

**DOI:** 10.3389/fsurg.2024.1409692

**Published:** 2024-08-16

**Authors:** Daniel Friel Leach, Srivikram Margam S, Aaron Gustin, Paul J. Gustin, Mohamad N. Jajeh, Yhana C. Chavis, Kristin V. Walker, Joshua S. Bentley

**Affiliations:** ^1^ Department of Radiation Oncology, University of Virginia Health, Charlottesville, VA, United States; ^2^ Research, Alabama College of Osteopathic Medicine, Dothan, AL, United States; ^3^ Neurological Surgery, Carle BroMenn Medical Center, Normal, IL, United States; ^4^ Internal Medicine, Southeast Health, Dothan, AL, United States; ^5^ Cerebrovascular and Endovascular Neurosurgery, Southeast Health, Dothan, AL, United States

**Keywords:** moyamoya disease, ischemic stroke, cervicocerebral catheter angiography, pre-operative Suzuki angiography staging, surgical revascularization, post-operative Matsushima grade

## Abstract

Moyamoya disease (MMD) is a chronic, occlusive cerebrovasculopathy typified by progressive steno-occlusive disease of the intracranial internal carotid arteries (ICAs) and their proximal branches. Moyamoya syndrome (MMS) categorizes patients with characteristic MMD plus associated conditions. As such, the most usual presentations are those that occur with cerebral ischemia, specifically transient ischemic attack, acute ischemic stroke, and seizures. Hemorrhagic stroke, headaches, and migraines can also occur secondary to the compensatory growth of fragile collateral vessels propagated by chronic cerebral ischemia. While the pathophysiology of MMD is unknown, there remain numerous clinical associations including radiation therapy to the brain, inherited genetic syndromes, hematologic disorders, and autoimmune conditions. We describe the case of a 31-year-old woman who presented with recurrent ischemic cerebral infarcts secondary to rapidly progressive, bilateral MMD despite undergoing early unilateral surgical revascularization with direct arterial bypass. She had numerous metabolic conditions and rapidly decompensated, ultimately passing away despite intensive and aggressive interventions. The present case highlights that progression of moyamoya disease to bilateral involvement can occur very rapidly, within a mere 6 weeks, a phenomenon which has not been documented in the literature to our knowledge.

## Introduction

Moyamoya disease (MMD) entails stenosis or complete occlusion of the intracranial ICA with clinical presentation including ischemic and/or hemorrhagic stroke as well as bilateral involvement. MMD is differentiated from moyamoya syndrome (MMS) by the fact that the former typically has bilateral stenosis while the latter tends to be unilateral with vascular findings occurring in the presence of an underlying pathological process associated with cerebral vasculopathy, such as cranial irradiation, Down syndrome, neurofibromatosis type 1, sickle cell disease, and Grave's disease (1–4). Both are distinguished from more common causes of vessel stenosis, such as atherosclerosis and vasculitis, as MMD patients rarely have atheromatous plaques observed in their arterial walls (1). There is a 6%–12% risk of developing MMD in the setting of an afflicted first-degree relative. It is more common in ethnic Asians, particularly in patients with Korean or Japanese heritage (2). MMD is a major cause of ischemic brain injury, seizures, and hemorrhagic stroke in pediatric populations with a bimodal age distribution in children and young adults (5–7).

MMD typically involves the terminal portions of the ICA and/or the proximal parts of the anterior cerebral artery (ACA) or middle cerebral artery (MCA), thus primarily affecting the anterior circulation. Posterior circulation involvement (i.e., posterior cerebral artery, basilar artery) occurs less frequently. Ischemia to the brain parenchyma can lead to a vascular response in the form of recruitment of collateral vessels (1). Though previously believed to be a process of angiogenesis with entirely new vessels, recent research indicates that existing vessels dilate and hypertrophy in response to the ischemia (1, 8). Specifically, the lenticulostriate or thalamoperforating arteries tend to be recruited. The enlarged collateral blood vessels give the syndrome its characteristic name “moyamoya”, translating to “puff of smoke”, which characterizes blood vessel appearance on cerebral angiography (1). Compared to normal samples, histological specimens from patients with MMD demonstrate relative thickening of the tunica intima secondary to smooth muscle cell hyperplasia (9), thinning of the tunica media with abnormal duplication and coiling of the internal elastic lamina (10), and micro-aneurysmal formation which could explain the relatively high incidence of hemorrhage in MMD patients (11). With this constellation of findings, histopathologic analysis of surgical specimens could potentially be used to confirm the diagnosis of MMD (12). Furthermore, apoptosis mediated by an activated caspase seems to be responsible for the pathological changes seen in specimens rather than necrosis, thus pointing towards a potential autoimmune mechanism for MMD (13, 14) with one study finding six autoantibodies associated with MMD (15). Additional studies have found increased autoimmunity in patients with MMD particularly those with unilateral involvement (16, 17). Moreover, autoimmune conditions including autoimmune thyroiditis (18), systemic lupus erythematosus (19), Sjogren's syndrome, neuromyelitis optica (20), and multiple sclerosis (21) have been clinically linked to MMD in case reports.

Diagnosis is made by computed tomography angiography (CTA), magnetic resonance angiography (MRA), or catheter-based digital subtraction angiography (DSA), which is the gold-standard. With disease advancement, vessels are recruited from the leptomeninges and eventually form anastomoses with the external carotid artery (ECA), a phenomenon coined as “ICA-to-ECA conversion” by some authors. It is described by Suzuki angiography staging and, more recently, Berlin moyamoya grading (1, 6, 12). Treatment consists of surgical revascularization via direct bypass (vessel-to-vessel), indirect synangiosis (vessel-to-brain parenchyma), or combined approaches to promote development of collateral circulation to the brain the results of which are quantified by Matsushima grade. Surgical revascularization aims to decrease the risk of ischemic or hemorrhagic stroke. However, pre-operative Suzuki stage, collateral score, degree of stenosis, and post-operative Matsushima grade appear to have mixed clinical correlation to disease presentation and progression (5, 22).

Progression of MMD to bilateral involvement can occur very rapidly, within just 6 weeks in the present case. Furthermore, disease progression occurred despite early unilateral surgical revascularization with direct arterial bypass, which is typically a positive prognosticator.

## Case presentation

A 31-year-old female with a past medical history of myocardial infarction (MI) and coronary artery disease status post multiple percutaneous coronary interventions for re-occlusive disease, mostly bed-bound following her MI, presented with a two-to-three day history of right sided upper and lower extremity numbness and right leg weakness. Her past medical history was also notable for poorly controlled insulin-dependent type 2 diabetes, hypertension, hyperlipidemia, tobacco use, and obesity raising concern for acute stroke. Neurological examination was remarkable for right cranial nerve five and seven palsies, bilateral hemiparesis more pronounced on the right, right hemisensory loss, and impaired cognition with a National Institutes of Health Stroke Scale of 7.

**Figure 1:**
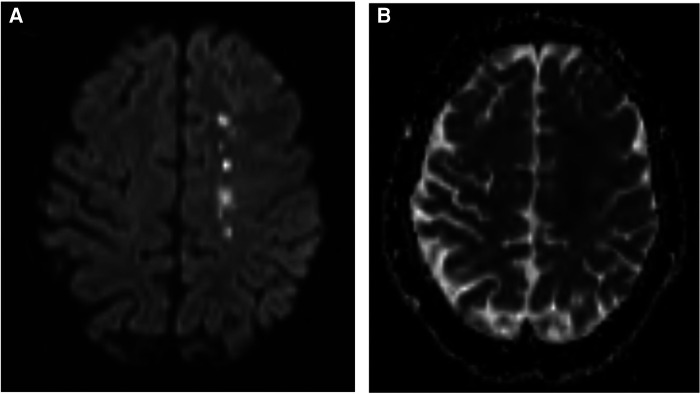
Preoperative MRI brain without contrast, DWI **(A)** and ADC **(B)** sequences, demonstrating acute ischemia involving the left frontal lobe at the ACA-MCA border zone.

**Figure 2:**
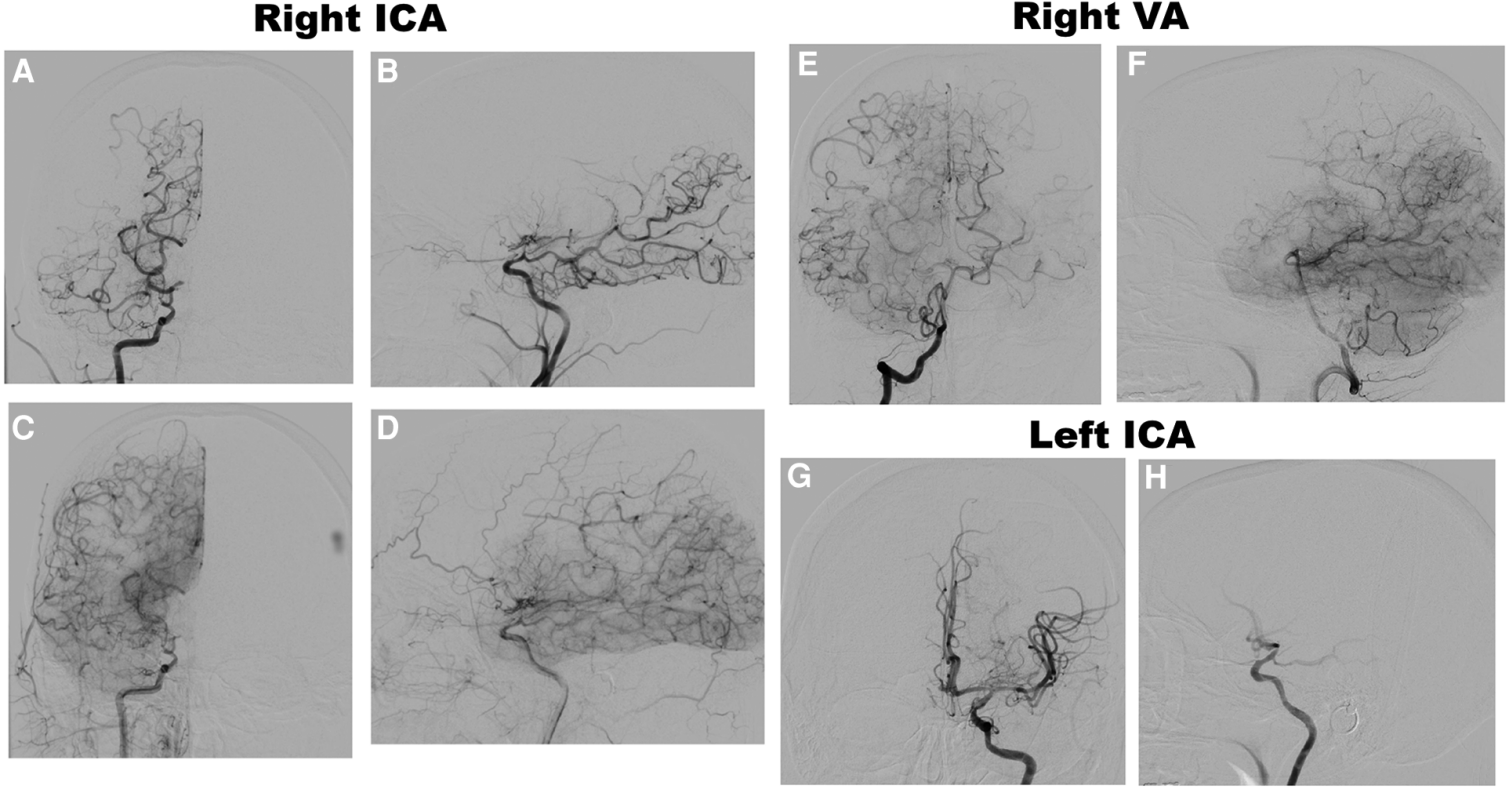
Preoperative DSA of the right ICA, anteroposterior (AP) **(A)** and lateral **(B)** views, early arterial phase, demonstrating occlusion of the right distal intracranial ICA distal to the pCoA, and AP **(C)** and lateral **(D)** views, late arterial phase, demonstrating leptomeningeal collateralization. DSA of the right VA, AP **(E)** and lateral **(F)** views, late arterial phase, demonstrating collateralization to the MCA and ACA territories. DSA of the left ICA, AP **(G)** and lateral **(H)** views, early arterial phase, demonstrating moderate stenosis of the distal ICA and proximal MCA and ACA with lenticulostriate collateralization.

Magnetic resonance imaging (MRI) brain without contrast showed acute bilateral ischemic infarcts in the left ACA-MCA border zone, involving the left paramedian frontal lobe, left periatrial white matter, and lateral right frontal lobe (Figure 1). Additional findings included a small chronic right parietal lobe infarct and mild microvascular ischemic disease of the periventricular and subcortical white matter. A transthoracic echocardiogram (TTE) did not identify a mural thrombus or any other cardioembolic phenomena. Bubble study was negative for intracardiac or intrapulmonary shunting. CTA of the head and neck and subsequent catheter-based cervicocerebral DSA demonstrated occlusion of the right intracranial ICA just distal to the posterior communicating artery (PCoA) with collateralization to the right MCA and ACA territories through posterior circulation collaterals, consistent with Suzuki stage 6, as well as stenosis of the left distal intracranial ICA distal to the PCoA, and left proximal MCA and ACA (Figure 2), consistent with Suzuki stage 3.

**Figure 3:**
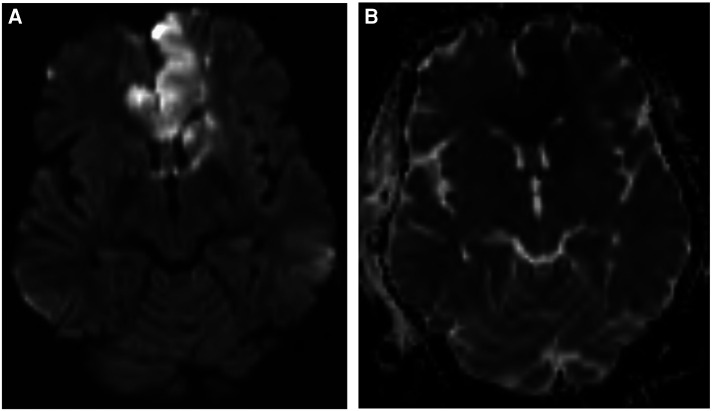
Postoperative MRI brain without contrast, DWI **(A)** and ADC **(B)** sequences, demonstrating acute ischemia involving the bilateral ACA and MCA territories.

**Figure 4:**
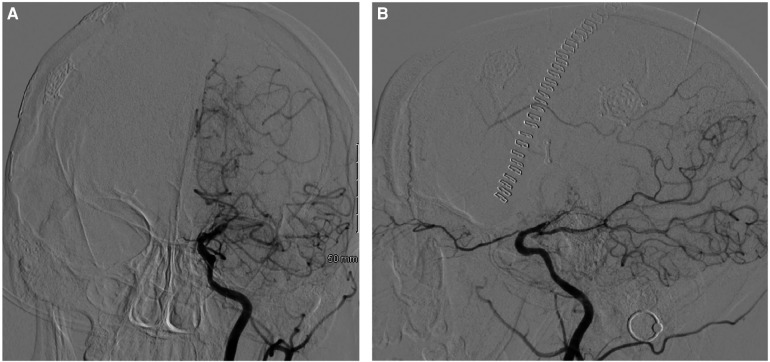
Postoperative DSA of the left ICA, AP **(A)** and lateral **(B)** views, demonstrating occlusion of the left distal intracranial ICA distal to the pCoA.

The patient was started on dual antiplatelet therapy and continued on a statin given MRI findings of bilateral stroke. She subsequently underwent elective direct revascularization with right superficial temporal artery-middle cerebral artery (STA-MCA) bypass. On postoperative day one, CT head without contrast demonstrated expected postoperative changes. On postoperative day two, she had new right hemiparesis and decreased speech output. Repeat CT head without contrast re-demonstrated expected postoperative changes. Neurology was consulted, levetiracetam levels were found to be therapeutic, and electroencephalography demonstrated nonspecific encephalopathy without epileptiform activity. MRI brain without contrast demonstrated new acute infarcts involving the bilateral ACA and MCA territories (Figure 3). CTA head and neck and subsequent catheter-based cervicocerebral DSA demonstrated occlusion of the left distal intracranial ICA distal to the PCoA with collateralization to the left MCA and ACA territories through posterior circulation collaterals (Figure 4), consistent with rapid progression to Suzuki stage 6 within 6 weeks of initial diagnosis.

A left STA-MCA bypass was scheduled to complete bilateral revascularization, but prior to this, she developed acute hypoxic respiratory failure necessitating intubation and initiation of mechanical ventilation. CT chest, abdomen, and pelvis demonstrated complete opacification of the left lung, with a polymicrobial sputum culture indicating aspiration pneumonia, for which the patient completed additional courses of broad-spectrum antibiotics. The patient also subsequently underwent bronchoscopy to alleviate mucous plugging. However, the patient unfortunately suffered three cardiac arrest events during the procedure with rapid return of spontaneous circulation after chest compressions and epinephrine. She was unable to tolerate being weaned off mechanical ventilation and underwent tracheostomy and percutaneous endoscopic gastrostomy tube placement.

Following the patient's cardiac arrest events, her neurologic exam was found to be poor. She was a Glasgow Coma Score 3 T with pupils fixed and dilated and exhibited no brainstem reflexes, no responses to painful stimuli, and was not breathing over the ventilator consistent with brain death. Consequently, the family decided to pursue comfort measures only and the patient expired shortly thereafter.

## Discussion

In the present case, diagnosis was determined by DSA, MRI, and CTA which together showed (1) steno-occlusion of the intracranial ICAs bilaterally and (2) vascular collateralization near the steno-occlusive lesions with right more prominent than left. Diagnosis of MMD can also be determined by MRI or MRA alone, obviating the need for DSA (12). However, the MRI studies here did not localize the steno-occlusive lesions of the ICA nor reveal abnormal vascular networks so acute ischemic stroke secondary to atheroembolism would remain in the differential diagnosis based on MRI alone; cardioembolism was ruled out by a negative TTE.

Unilateral revascularization was selected as DSA evidenced advanced MMD on the right side (Suzuki stage 6) and the patient exhibited left-sided hemiparesis on neurologic exam; she likely did not report left-sided symptoms due to the degree of bilateral frontal lobe infarction. While the patient did exhibit clinical and radiographic signs of left-sided vasculopathy (Suzuki stage 3) on her initial presentation, and surgical revascularization is recommended for patients with at least Suzuki stage 2 disease thus providing an indication for bilateral revascularization here, patients with MMD are at high risk for anesthetic complications due to tenuous blood supply (23). With her metabolic conditions conveying additional perioperative risk, bilateral revascularization was judged to entail less risk through successive operations by reducing overall time under anesthesia.

Although the patient underwent right STA-MCA bypass to treat ipsilateral disease soon after diagnosis, the rapid progression of her disease with advanced bilateral involvement resulted in a second ischemic stroke, the complications of which resulted in her death before collateralization from the right-sided bypass could mature or left-sided surgical revascularization could be performed. Her young age entailed an increased risk of MMD progression to bilateral involvement (24). Moreover, her advanced Suzuki stage (greater than 3), female sex, and subsequent symptomatic progression of her left sided MMD likely increased her risk of additional stroke in the setting of classic stroke risk factors, namely hypertension and hyperlipidemia (22, 25). Her metabolic syndrome entailed increased cardiovascular mortality (26) which occurred late after repeated hospital courses. Although there is up to a 30% rate of progression with unilateral MMD (27), there is no consensus on whether bilateral surgical revascularization is required for bilateral MMD with one case series recommending unilateral revascularization to the symptomatic side based on their findings (28) as performed in our case. A case described by Li et al. demonstrated that unilateral bypass was sufficient despite bilateral involvement with no recurrent ischemic or hemorrhagic stroke within a 3 year follow-up period (29). However, in a retrospective study by Yu et al, bilateral revascularization appeared more effective in preventing rebleeeding in hemorrhagic MMD than unilateral revascularization, although there was no difference in functional outcomes between patients in the bilateral revascularization and unilateral revascularization groups (30).

Three surgical revascularization techniques are appropriate in the treatment of MMD: direct (i.e., bypass), indirect (i.e., synangiosis), and combined revascularization. While endovascular treatment is the standard of care for MMD-associated aneurysms (31–33), it is a controversial treatment modality for steno-occlusive lesions as the procedure may propagate the vasculitis-like angiopathy with concentric stenosis seen in MMD. However, some studies have reported endovascular treatment can be applied to MMD although it remains technically challenging (34). Generally, surgical revascularization is recommended as it improves functional outcomes and, in hemorrhagic MMD, reduces time to rebleed on the operated side relative to conservative treatment (30).

With direct revascularization, STA-MCA bypass is the most common as it provides early revascularization and addresses the MCA territory as well as the ACA territory via leptomeningeal anastomoses. As such, it was selected in this case. However, STA-ACA, STA-PCA, and occipital artery-PCA anastomosis may be performed to address the ACA and PCA territories discretely. Radial artery or saphenous vein grafts may be utilized for intermediate- or high-flow bypass, respectively, but entail potential risk of reperfusion hemorrhage due to hyperperfusion syndrome (6, 35). Direct revascularization has a robust evidence base demonstrating efficacy in reducing hemorrhagic events in MMD patients (35). While the same evidence is not currently established for ischemic MMD, meta-analyses indicate that the direct techniques are highly effective at secondary prevention of ischemic events including ischemic stroke in adults. Aside from the arduous demands of the surgery itself, direct bypass requires the appropriate quantity of donor and recipient vessels. Consequently, the rate of patency of the direct bypass technique is significantly lower in children than adults. This combined with the fact that there is an age-dependent decline in cerebrovascular plasticity in patients with MMD points to indirect revascularization techniques as a more effective strategy in children (6) although one study found durable revascularization with pial synangiosis in adults (36).

Indirect revascularization is dependent on angiogenesis to the cortical surface from transplanted blood vessels (typically the STA), dura, galea, temporal muscle grafts (i.e., encephalo-myo-synangiosis referred to as EMS), or omentum. A multifaceted transplant approach, known as encephalo-duro-arterio-synangiosis (EDAS), entails suturing a branch of the STA in between two leaves of dura. Pial synangiosis is a variant of EDAS whereby the STA is directly sutured to the pia mater followed by opening of the arachnoid to facilitate ingrowth of new vessels (37). Additional multifaceted indirect approaches include encephalo-arterio-synagiosis with cranioplasty (38), encephalo-duro-myo-synangiosis (39), encephalo-duro-pericranio-synangiosis (40), and encephalo-duro-myoarterio-pericranio-synangiosis (41). Sternocleidomastoid EMS using a tunneled muscle flap has been successfully employed which facilitated revascularization, particularly in the parietal and occipital lobes, in a patient with MMD refractory to bilateral arterial bypass and temporalis EMS (42). Multiple burr-hole surgery is another unique indirect method which appears to provide good collateralization with good symptomatic control and low peri-operative risk (43, 44). In general, the revascularization process is slower with indirect compared to direct approaches due to the reliance on angiogenesis thus entailing a temporarily higher risk of stroke within a 6 to 12-month period. However, indirect approaches are technically less demanding and provide robust, long-term revascularization potentially beyond the target vascular territory (34, 35, 45).

Combined revascularization is typically selected when direct revascularization fails with the objective of combining the early hemodynamic improvement seen in direct bypass with the delayed improvement of the indirect technique. A case described by Couldwell et al. showed the success of combined revascularization for the rare presentation of cerebellar hemorrhagic stroke in MMD, which achieved good revascularization and premorbid functional status with normal cognitive function over a 2 year follow-up period (46). However, a case described by Zhang et al. highlighted intraventricular hemorrhage secondary to postoperative hyperperfusion syndrome as an acute consequence of combined revascularization (34). Management of such postoperative sequelae, particularly rebleeds, is unclear. Quantitative analysis of the flow in patients undergoing combined revascularization suggests that there is more than a simple additive effect between direct and indirect techniques with some earlier studies indicating that direct bypass tends to decline by the 6-month mark while the indirect collaterals begin to compensate for this decline concurrently. While the eventual hemodynamic result is unpredictable, recent studies have shown that roughly half of children and adults still develop sufficient direct and indirect bypasses as the result of the combined revascularization (6).

No surgical technique has demonstrated superiority, each with certain risks and benefits, but all improve neurological outcomes.

## Conclusions

The present case highlights rapid progression of MMD disease to bilateral involvement within just 6 weeks despite early unilateral surgical revascularization, an aggressive presentation not previously described. Thus, it is important for clinicians to be aware that patients with MMD can rapidly deteriorate and select an appropriate surgical intervention, each with innate risks and benefits, as quickly as clinically feasible to provide patients with the greatest chance of a positive outcome. However, as moyamoya disease is rare with the described case representing a unique presentation, further studies with greater patient populations are required to determine optimal treatment and clinical outcomes.

## Data Availability

The original contributions presented in the study are included in the article/Supplementary Material, further inquiries can be directed to the corresponding author.

## References

[1] Berry JA Cortez V Toor H Saini H Siddiqi J. Moyamoya: an update and review. Cureus. (2020) 12(10):e10994. 10.7759/cureus.10994 33209550 PMC7667711

[2] Phi JH Wang KC Lee JY Kim SK. Moyamoya syndrome: a window of moyamoya disease. J Korean Neurosurg Soc. (2015) 57(6):408–14. 10.3340/jkns.2015.57.6.408 26180607 PMC4502236

[3] Nakamura H Sato K Yoshimura S Hayashi Y Izumo T Tokunaga Y. Moyamoya disease associated with Graves’ disease and down syndrome: a case report and literature review. J Stroke Cerebrovasc Dis. (2021) 30(1):105414. 10.1016/j.jstrokecerebrovasdis.2020.105414 33130479

[4] Hayashi K Morofuji Y Horie N Izumo T. A case of neurofibromatosis type 1 complicated with repeated intracerebral hemorrhage due to Quasi-Moyamoya disease. J Stroke Cerebrovasc Dis. (2015) 24(5):e109–113. 10.1016/j.jstrokecerebrovasdis.2014.12.029 25804563

[5] Rosi A Riordan CP Smith ER Scott RM Orbach DB. Clinical status and evolution in moyamoya: which angiographic findings correlate? Brain Commun. (2019) 1(1):fcz029. 10.1093/braincomms/fcz029 32954269 PMC7425301

[6] Acker G Fekonja L Vajkoczy P. Surgical management of moyamoya disease. Stroke. (2018) 49(2):476–82. 10.1161/STROKEAHA.117.018563 29343587

[7] Hannon KE. Pial synangiosis for treatment of moyamoya syndrome in children. AORN J. (1996) 64(4):540–54. quiz 557–60. 10.1016/S0001-2092(06)63621-1 8893961

[8] Bedini G Blecharz KG Nava S Vajkoczy P Alessandri G Ranieri M Vasculogenic and angiogenic pathways in moyamoya disease. Curr Med Chem. (2016) 23(4):315–45. 10.2174/092986732304160204181543 26861126

[9] Fukui M Kono S Sueishi K Ikezaki K. Moyamoya disease. Neuropathology. (2000) 20(s1):61–4. 10.1046/j.1440-1789.2000.00300.x 11037190

[10] Takagi Y Kikuta K Nozaki K Hashimoto N. Histological features of middle cerebral arteries from patients treated for moyamoya disease. Neurol Med Chir (Tokyo). (2007) 47(1):1–4. 10.2176/nmc.47.1 17245006

[11] Yamashita M Tanaka K Matsuo T Yokoyama K Fujii T Sakamoto H. Cerebral dissecting aneurysms in patients with moyamoya disease. Report of two cases. J Neurosurg. (1983) 58(1):120–5. 10.3171/jns.1983.58.1.0120 6847898

[12] Fujimura M Tominaga T. Diagnosis of moyamoya disease: international standard and regional differences. Neurol Med Chir (Tokyo). (2015) 55(3):189–93. 10.2176/nmc.ra.2014-0307 25739428 PMC4533332

[13] Takagi Y Kikuta K Sadamasa N Nozaki K Hashimoto N. Caspase-3-dependent apoptosis in middle cerebral arteries in patients with moyamoya disease. Neurosurgery. (2006) 59(4):894–900. discussion 900–901. 10.1227/01.NEU.0000232771.80339.15 17038954

[14] Asselman C Hemelsoet D Eggermont D Dermaut B Impens F. Moyamoya disease emerging as an immune-related angiopathy. Trends Mol Med. (2022) 28(11):939–50. 10.1016/j.molmed.2022.08.009 36115805

[15] Sigdel TK Shoemaker LD Chen R Li L Butte AJ Sarwal MM Immune response profiling identifies autoantibodies specific to moyamoya patients. Orphanet J Rare Dis. (2013) 8:45. 10.1186/1750-1172-8-45 23518061 PMC3648437

[16] Santoro JD Lee S Wang AC Ho E Nagesh D Khoshnood M Increased autoimmunity in individuals with down syndrome and moyamoya disease. Front Neurol. (2021) 12:724969. 10.3389/fneur.2021.724969 34566869 PMC8455812

[17] Chen JB Liu Y Zhou LX Sun H He M You C. Increased prevalence of autoimmune disease in patients with unilateral compared with bilateral moyamoya disease. J Neurosurg. (2016) 124(5):1215–20. 10.3171/2015.4.JNS142936 26406790

[18] Lin YH Huang H Hwang WZ. Moyamoya disease with Sjogren disease and autoimmune thyroiditis presenting with left intracranial hemorrhage after messenger RNA-1273 vaccination: a case report. Medicine (Baltimore). (2022) 101(6):e28756. 10.1097/MD.0000000000028756 35147099 PMC8830843

[19] Das S Dubey S Pandit A Ray BK. Moyamoya angiopathy unmasking systemic lupus erythematosus. BMJ Case Reports CP. (2021) 14(1):e239307. 10.1136/bcr-2020-239307 PMC7843324 33504534

[20] Asai Y Nakayasu H Fusayasu E Nakashima K. Moyamoya disease presenting as thalamic hemorrhage in a patient with neuromyelitis optica and Sjögren’s syndrome. J Stroke Cerebrovasc Dis. (2012) 21(7):619.e7–9. 10.1016/j.jstrokecerebrovasdis.2011.01.003 21571549

[21] Koul P Patel A Chaudhry F Steinklein J Harel A. A patient with concurrent multiple sclerosis and moyamoya disease. Mult Scler Relat Disord. (2021) 54:103151. 10.1016/j.msard.2021.103151 34293702

[22] Zhao M Zhang D Wang S Zhang Y Wang R Zhao J. Transient ischemic attack in pediatric patients with moyamoya disease: clinical features, natural history, and predictors of stroke. Pediatr Neurol. (2017) 75:48–54. 10.1016/j.pediatrneurol.2017.06.020 28778481

[23] Zipfel GJ Fox DJ Rivet DJ. Moyamoya disease in adults: the role of cerebral revascularization. Skull Base. (2005) 15(1):27–41. 10.1055/s-2005-868161 16148982 PMC1151702

[24] Smith ER Scott RM. Progression of disease in unilateral moyamoya syndrome. Neurosurg Focus. (2008) 24(2):E17. 10.3171/FOC/2008/24/2/E17 18275294

[25] Hirano Y Miyawaki S Imai H Hongo H Ohara K Dofuku S Association between the onset pattern of adult moyamoya disease and risk factors for stroke. Stroke. (2020) 51(10):3124–8. 10.1161/STROKEAHA.120.030653 32867597

[26] Nguyen NT Nguyen TN Nguyen KM Tran HPN Huynh KLA Hoang SV. Prevalence and impact of metabolic syndrome on in-hospital outcomes in patients with acute myocardial infarction: a perspective from a developing country. Medicine (Baltimore). (2023) 102(45):e35924. 10.1097/MD.0000000000035924 37960714 PMC10637448

[27] Church EW Bell-Stephens TE Bigder MG Gummidipundi S Han SS Steinberg GK. Clinical course of unilateral moyamoya disease. Neurosurgery. (2020) 87(6):1262–8. 10.1093/neuros/nyaa284 32710766

[28] Seol HJ Wang KC Kim SK Lee CS Lee DS Kim IO Unilateral (probable) moyamoya disease: long-term follow-up of seven cases. Childs Nerv Syst. (2006) 22(2):145–50. 10.1007/s00381-005-1234-1 16220301

[29] Li X Zhao N Yang PZ. One sided bypass for bilateral moyamoya disease, a case report and review of the literatures. Int J Surg Case Rep. (2016) 22:15–8. 10.1016/j.ijscr.2016.03.015 27016648 PMC4844669

[30] Yu S Zhang N Liu J Li C Qian S Xu Y Surgical revascularization vs. Conservative treatment for adult hemorrhagic moyamoya disease: analysis of rebleeding in 322 consecutive patients. Neurosurg Rev. (2022) 45(2):1709–20. 10.1007/s10143-021-01689-w 34859335

[31] Zhang L Xu K Zhang Y Wang X Yu J. Treatment strategies for aneurysms associated with moyamoya disease. Int J Med Sci. (2015) 12(3):234–42. 10.7150/ijms.10837 25678840 PMC4323361

[32] Jiang H Ni W Lei Y Li Y Gu Y. Combined extracranial-intracranial bypass surgery with stent-assisted coil embolization for moyamoya disease with a ruptured wide-necked basilar trunk aneurysm: a case report. Turk Neurosurg. (2015) 25(1):180–5. 10.5137/1019-5149.JTN.10043-13.0 25640568

[33] Kagawa K Ezura M Shirane R Takahashi A Yoshimoto T. Intraaneurysmal embolization of an unruptured basilar tip aneurysm associated with moyamoya disease. J Clin Neurosci. (2001) 8(5):462–4. 10.1054/jocn.2000.0806 11535021

[34] Zhang X Xiao W Zhang Q Xia D Gao P Su J Progression in moyamoya disease: clinical features, neuroimaging evaluation, and treatment. Curr Neuropharmacol. (2022) 20(2):292–308. 10.2174/1570159X19666210716114016 34279201 PMC9413783

[35] Nguyen VN Parikh KA Motiwala M Erin Miller L Barats M Milton C Surgical techniques and indications for treatment of adult moyamoya disease. Front Surg. (2022) 9:966430. 10.3389/fsurg.2022.966430 36061058 PMC9437590

[36] Lin N Aronson JP Manjila S Smith ER Scott RM. Treatment of moyamoya disease in the adult population with pial synangiosis. J Neurosurg. (2014) 120(3):612–7. 10.3171/2013.11.JNS131027 24405066

[37] Scott RM. Surgical techniques in moyamoya vasculopathy. Tricks of the trade by Peter Vajkoczy (2019) 206 pp, 471 illustrations hardback ISBN: 9783131450616 (eBook, eISBN 978-3-13-147081-2) Thieme Publishers New York/Stuttgart. Acta Neurochir. (2020) 162(8):1839. 10.1007/s00701-020-04365-y

[38] Kato N Kakizaki S Hirokawa Y Michishita S Ishii T Terao T Encephalo-arterio-synangiosis with cranioplasty after treatment of acute subdural hematoma associated with subcortical hemorrhage due to unilateral moyamoya disease. Case Rep Neurol Med. (2023) 2023:1787738. 10.1155/2023/1787738 36704418 PMC9873458

[39] Shen W Xu B Li H Gao X Liao Y Shi W Enlarged encephalo-duro-myo-synangiosis treatment for moyamoya disease in young children. World Neurosurg. (2017) 106:9–16. 10.1016/j.wneu.2017.06.088 28645592

[40] Yamashina M Inaji M Hara S Tanaka Y Kaneoka A Nariai T Encephalo-duro-pericranio-synangiosis for the treatment of moyamoya disease with posterior cerebral artery lesions. World Neurosurg. (2023) 175:e678–85. 10.1016/j.wneu.2023.04.006. 37030475

[41] July J. A case report of moyamoya disease in children treated with encephalo-duro-myo-arterio-pericranial synangiosis. Med J Indonesia. (2021) 30(3):228–31. 10.13181/mji.cr.204452

[42] Chiarelli PA Patel AP Lee A Chandra SR Sekhar LN. Sternocleidomastoid encephalomyosynangiosis for treatment-resistant moyamoya disease. Oper Neurosurg (Hagerstown). (2019) 17(1):E23–8. 10.1093/ons/opy234 30169838

[43] Mirone G Cicala D Meucci C d’Amico A Santoro C Muto M Multiple burr-hole surgery for the treatment of moyamoya disease and quasi-moyamoya disease in children: preliminary surgical and imaging results. World Neurosurg. (2019) 127:e843–55. 10.1016/j.wneu.2019.03.282 30954732

[44] Kamada C Hirano T Mikami T Komatsu K Suzuki H Tsushima S Additional revascularization using multiple burr holes for PCA involvement in moyamoya disease. J Stroke Cerebrovasc Dis. (2021) 30(8):105852. 10.1016/j.jstrokecerebrovasdis.2021.105852 34015559

[45] Fiaschi P Scala M Piatelli G Tortora D Secci F Cama A Limits and pitfalls of indirect revascularization in moyamoya disease and syndrome. Neurosurg Rev. (2021) 44(4):1877–87. 10.1007/s10143-020-01393-1 32959193 PMC8338852

[46] Couldwell MW Cheshier S Taussky P Mortimer V Couldwell WT. Right frontotemporal craniotomy for ECA-to-MCA direct and indirect bypass and occipital artery indirect bypass to the posterior circulation: case report. J Neurosurg Pediatr. (2020) 27(2):180–4. 10.3171/2020.7.PEDS20181 33254140

