# Corrigendum: Case Report: A rare presentation of rapidly progressive moyamoya disease refractory to unilateral surgical revascularization

**DOI:** 10.3389/fsurg.2024.1550675

**Published:** 2025-01-10

**Authors:** Daniel F. Leach, Srivikram Margam S, Aaron J. Gustin, Paul J. Gustin, Mohamad N. Jajeh, Yhana C. Chavis, Kristin V. Walker, Joshua S. Bentley

**Affiliations:** ^1^ Department of Radiation Oncology, University of Virginia Health, Charlottesville, VA, United States; ^2^ Research, Alabama College of Osteopathic Medicine, Dothan, AL, United States; ^3^ Neurological Surgery, Carle BroMenn Medical Center, Normal, IL, United States; ^4^ Internal Medicine, Southeast Health, Dothan, AL, United States; ^5^ Cerebrovascular and Endovascular Neurosurgery, Southeast Health, Dothan, AL, United States

**Keywords:** moyamoya disease, ischemic stroke, cervicocerebral catheter angiography, preoperative Suzuki angiography staging, surgical revascularization, post-operative Matsushima grade

**A Corrigendum on**
Case Report: A rare presentation of rapidly progressive moyamoya disease refractory to unilateral surgical revascularization By Leach III DF, Margam S S, Gustin AJ, Gustin PJ, Jajeh MN, Chavis YC, Walker KV, Bentley JS. (2024). Front. Surg. 11:1409692. doi: 10.3389/fsurg.2024.1409692

**Incorrect Author Name**

In the published article, an author name was incorrectly written as Aaron Gustin. The correct spelling is Aaron J. Gustin. An author name was incorrectly written as Srivikram Margam. The correct spelling is Srivikram Margam S.

The authors apologize for this error and state that this does not change the scientific conclusions of the article in any way. The original article has been updated.

